# Supplementary material for: Effects of different marination conditions on the physico-chemical and microbiological quality of anchovy (Engraulis encrasicolus) fillets inoculated with Morganella psychrotolerans during cold storage
Source: PLoS One. 2024 Jul 8;19(7):e0302038. doi: 10.1371/journal.pone.0302038 (PMC11230567; doi:10.1371/journal.pone.0302038)
Supplement: Attachment — Submitted filename: response to reviewers.docx [file pone.0302038.s002.docx]

**Answer to Reviewers' comments**

Dear Editor,

Our manuscript, entitled: **“Effects of different marination conditions on the physico-chemical and microbiological quality of anchovy (*Engraulis encrasicolus*) fillets inoculated with *Morganella psychrotolerans* during cold storage”** was revised carefully according to the comments from reviewers. All changes made in the text were track changes and a clean copy was also created. We would like to thank to the reviewers for their kind and useful comments for the contribution of paper.

Below you will find our point- by- point responses to the reviewers’ comments (**Q**: questions

**R**: response)

We hope that the revised paper will meet the journal’s standard.

Thank you very much again for your attention to our paper．

**Reviewer 1**

**Q:** Too many spelling and grammatical errors in the text.

**R:** Thank you for your suggestions. Manuscript edited carefully and all spelling and gramatical errors were corrected.

**Q:** The title is not written with due care and the scientific names in the text are not written with due care.

**R:** Thank you for the comment. Title was edited carefully and scientific names were corrected through the manuscript.

**Q:** References are generally very old. For the 2024 article, too many old references (2004,2012,2011,2014,2013) should be supplemented with new references.

**R:** thank you very much for this valuable suggestion. References were revised and updated.

**Q:** Lines 125, 131 and 135 are not referenced.

**R:** Thank you for the carefull review. References were added.

**Q:** There is no information on why the acid levels, time and inputs selected in the marination procedure were chosen. In the analysis periods, for the marinades matured in 21 days, why the 0,3 and 15th day analyzes were made and on the basis of which parameter the marination was completed.

**R:** Thank you very much for this valuable comment. The marination parameters were chosen according to the pre-studies which were conducted before the main study. Different acetic acid and salt concentrations were applied to the anchovy fillets and evaluated in terms of sensorial parameters. Then the most selected concentrations were chosen. Additionally, in the literature mostly those concentrations are applied. Maturation time is also based on the sensory evaluation of pre-study. These informations were not included in the manuscript in order to avoid too much writing.

**Q:** Although microbial analyzes are appropriate in the analysis, the lack of salinity and acidity analyzes despite sensory evaluation is a serious deficiency. Again, since it is an oily fish, PV and TBA are correct analyses, but since the goal in marinating is to reduce acidity and activate protease enzymes, it would be meaningful to have TVB-N data in the analysis.

**R:** Thank you very much for valuable suggestion. Unfortunately our infrastructure were not suitable and the budget of the project were not enough for adding additional analyses like TVB-N.

**Q:** In the discussion part, especially pH, lactic acid, coliform and total bacteria results should be discussed with a holistic approach. The role of bacteria dominating each other and the role of pH in this process should be explained.

R: thank you for this suggestion. These sections were revised carefully and necessary information was given.

**Reviewer 2**

**Q:** I recommend that the authors shorten the abstract.

**R:** Thank you for the suggestion. Abstract was shortened and edited carefully.

**Q:** Check the sentence: What is 4.3?

**R:** Sentence was revised.

**Q:** Introduction: This section contains too much general information. I recommend shortening it. Also, provide at least 3 paragraphs of introduction instead of one.
- Hypothesis and objective: These parts in the introduction need to be improved and rewritten.
- Add a materials and methods section or an experimental section in the manuscript.

**R:** Thank you so much for this valuable comment. Introduction section was improved and rewritten. Additionally, suggested informations were given in this section.

**Q:** L 120: What are the conditions for obtaining anchovy fillets?

**R:** Thank you for this comment. Anchovy fillets were obtained from a local marked and details were included in the related section.

**Q:** L125: Did the authors follow any reference method for preparation? If so, include it as well.

**R:** Thank you very much for the suggestion. The preparation was conducted according to the German Collection of Microorganisms and Cell Culture (DSMZ, Braunschweig, Germany) specification as they suggested. Also reference was added.

**Q:** L132: Why 5 min? What is the temperature of the inoculum and the samples? Report it.

**R:** Thank you very much for the valuable question. The inocultaion procedure was conducted according to the method of Ucak et al. (2019) where they studies the same bacteria in marinated fish. Related reference were also added in this section.

**Q:** Marination process: There are many typos in this section, especially using symbols. Check it carefully. Similarly, check for typos throughout the manuscript.

**R:** This section was edited and suggested points were corrected.

**Q:** Provide a diagram or a flowchart for the sample preparation, inoculation, and marination.

R: Thank you for the valuable contribution. A flowchart was included in the material and method section.

**Q:** L143: Elaborate this section.

**R:** Section was elaborated and detailed.

**Q:** L164: Trained panelists? Experts in the field of what? Or did you train them specifically for this study? Include details as well.

**R:** Thank you for the valuable question. Details were given in the related section. The panelists were not trained but they were selected from people who have fish and marinated fish consumption habbit.

**Q:** L172: Why duplicate? Why not triplicate? Statistical analysis: This part is not clear. Please elaborate.

**R:** The section was edited. There was a mistake due to copy-paste from previous studies. All measurements were carried out in triplicate.

Q: Table 1: The columns that contain 0 values should use - or leave blank. If the authors use -, then provide a note below the table, as growth not found.

R: All tables were revised as suggested. We used ‘’-‘’ and put a note below the table as growth not found.

Q: Section *Morganella Psychrotolerans*: This section lacks discussion. Very few literature reviews are provided. Please elaborate it.

R: Thank you very much for the suggestion. This section was edited and more literature were added.

Q: I recommend splitting Table 1 into many, providing individual microorganism data and placing it below their discussion. For now, Table 1 is overloaded, not convenient to read, and overwhelming.

R: Thank you very much for the contribution. All tables were splitted and edited as recommended.

Q: A similar recommendation goes for Table 2 as well.

R: All tables were splitted and edited as recommended.

Q: Sensory analysis: Provide this part in figure format.

R: This part was rewritten and the table of this section converted to the figures.

Q: Conclusion: This part needs to be concise and targeting main points.

R: Thank you for the valuable comment. Conclusion section was edited and rewritten.

Q: Overall: This manuscript contains important data, which is useful, but the authors have written too much when it is not necessary. I recommend that the authors check thoroughly the writing style and include only the necessary information.

R: Thank you for your valuable time in commenting on the article. All comments have been responded and corrections have been made to the article.
